# An Inverse Dose Optimization Algorithm for Three-Dimensional Brachytherapy

**DOI:** 10.3389/fonc.2020.564580

**Published:** 2020-10-20

**Authors:** Xianliang Wang, Pei Wang, Bin Tang, Shengwei Kang, Qing Hou, Zhangwen Wu, Chengjun Gou, Lintao Li, Lucia Orlandini, Jinyi Lang, Jie Li

**Affiliations:** ^1^ Department of Radiation Oncology, Sichuan Cancer Hospital & Institute, School of Medicine, University of Electronic Science and Technology of China, Radiation Oncology Key Laboratory Of Sichuan Province, Chengdu, China; ^2^ Key Laboratory of Radiation Physics and Technology, Institute of Nuclear Science and Technology, Sichuan University, Chengdu, China

**Keywords:** brachytherapy, inverse optimization, cervical cancer, treatment planning system, dwell time

## Abstract

**Purpose:**

To investigate an implementation method and the results of an inverse dose optimization algorithm, Gradient Based Planning Optimization (GBPO), for three-dimensional brachytherapy.

**Methods:**

The GBPO used a quadratic objective function, and a dwell time modulation item was added to the objective function to restrict the dwell time variance. We retrospectively studied 4 cervical cancer patients using different applicators and 15 cervical cancer patients using the Fletcher applicator. We assessed the plan quality of GBPO by isodose lines for the patients using different applicators. For the 15 patients using the Fletcher applicator, we utilized dose-volume histogram (DVH) parameters of HR-CTV (D_100%_, V_150%_) and organs at risk (OARs) (D_0.1cc_, D_1cc_, D_2cc_) to evaluate the difference between the GBPO plans and the IPSA (Inverse Planning Simulated Annealing) plans, as well as the GBPO plans and the Graphic plans.

**Results:**

For the 4 patients using different applicators, the dose distributions are conformable. For the 15 patients using the Fletcher applicator, when the dwell time modulation factor (DTMF) is less than 20, the dwell time deviation reduces quickly; however, after the DTMF increased to 100, the dwell time deviation has no remarkable change. The difference in dosimetric parameters between the GBPO plans and the IPSA plans is not statistically significant (*P*>0.05). The GBPO plans have a higher D_100%_ (3.57 ± 0.36, 3.38 ± 0.34; *P*<0.01) and a lower V_150%_ (55.73 ± 4.06, 57.75 ± 3.79; *P*<0.01) than those of the Graphic plans. The differences in other DVH parameters are negligible between the GBPO plans and the Graphic plans.

**Conclusions:**

The GBPO plans have a comparable quality as the IPSA plans and the Graphic plans for the studied cervical cancer cases. The GBPO algorithm could be integrated into a three-dimensional brachytherapy treatment planning system after studying more sites.

## Introduction

Compared with external beam radiation therapy, brachytherapy has the characteristics of high dose near the source and rapid dose drop-off away from the source. In addition, because the applicator is implanted in the tumor region, brachytherapy reduces the dosimetric uncertainties caused by anatomical change and setup error. These advantages ensure the irreplaceable role of brachytherapy in radiotherapy (1).

At the present stage, image-guided three-dimensional (3D) brachytherapy is the mainstream method for brachytherapy. Dose optimization is a crucially important component of 3D brachytherapy treatment planning systems (TPSs). In general, dose optimization methods of 3D brachytherapy can be divided into forward optimization and inverse optimization. In a forward optimization process, a planner manually enters the dwelling weight/time or drags isodose lines based on the planner’s clinical experience to achieve a desirable dose distribution. The method of dragging isodose lines is called graphic optimization. In an inverse optimization process, a planner inputs the objectives and penalty weights of targets and organs at risk (OARs) based on the prescription dose and patient’s anatomy. Through a trial-and-error process, a satisfactory dose distribution can be generated by the inverse dose optimization system. Inverse optimization algorithms of brachytherapy, such as IPSA (Inverse Planning Simulated Annealing) and HIPO (Hybrid Inverse Planning Optimization), have been reported in literatures and implemented in 3D TPSs (2–5).

The inverse optimization algorithm of brachytherapy usually produces a plan with a large dwell time variation (6), which should be addressed for the following reasons: First, a location with a large dwell time is suspect to have a high dose. A high dose region should be avoided unless a tumor volume requires an inhomogeneous dose distribution. Second, the larger the dwell time variation, the greater the inhomogeneous dose distribution. An inhomogeneous dose distribution is more likely to be affected by source position uncertainties. Both IPSA and HIPO provide parameters that restrict dwell time variance: the Dwell Time Deviation Constraint (DTDC) and Dwell Time Gradient Restriction (DTGR) for IPSA and HIPO, respectively (4, 5). By adjusting these parameters, it is possible to obtain a favorable clinical plan for which the variation of the dwell time is considered acceptable.

The purpose of this paper is to investigate an in-house inverse brachytherapy optimization algorithm referred to as Gradient Based Planning Optimization or GBPO and a new method to restrict dwell time variance. We retrospectively studied a total of 4 cervical cancer patients using different applicators and 15 cervical cancer patients using the Fletcher applicator to evaluate the GBPO algorithm.

## Materials and Methods

### Dose Calculation

The dose calculation algorithm in this study was based on the AAPM TG-43 recommendation (7, 8). Since the implementation detail has been reported in the reference (9), only a brief introduction is included here. We calculated the dose of the *i*-th voxel, *D_i_*, through the formula given in Equation 1:

(1) $$D_{i}=\sum_{m=1}^{N_{M}}\sum_{N=1}^{N_{N}}d_{m,n}t_{m,n}$$

where *N_M_* is the total channel number, *N_N_* is the total dwell position number in the *m*-th channel, *d_m,n_* and *t_m,n_* are the dose rate contribution and the dwell weight, respectively, from the *n*-th dwell position in the *m*-th channel.

### Inverse Optimization

The GBPO optimization algorithm was implemented using the LBFGS (Limited memory Broyden Fletcher Goldberg Shanno) code, which is an optimization engine based on the gradient descent method (10, 11). The GBPO used a quadratic objective function, and we calculated the objective value *F* through the formula given in Equation 2:

(2) $$\begin{array}{l} F\left(t_{m,n}\right)={\sum_{i\in TAR}p_{TAR}\cdot H_{TAR}\left(D_{i}-D_{0,TAR}\right)\cdot \left(D_{i}-D_{0,TAR}\right)}^{2}\\ +\sum_{i\in OARs}p_{OARs}\cdot H_{OARs}(D_{i}-D_{0,OARs})\cdot {\left(D_{i}-D_{0,OARs}\right)}^{2}\\ +p_{SOU}\cdot \sum_{m=1}^{N_{M}}\sum_{n=1}^{N_{N}}\frac{1}{N_{N}}{\left(t_{m,n}-t_{m,\min}\right)}^{2} \end{array}$$

where *p_TAR_* is the penalty weight of the target; *H* (*D_i_* – *D*_0_) is a Heaviside function (12), and for a target it equals 0 if *D_i_* > *D*_0_ but 1 if *D_i_* ≤ *D*_0_; the value reverses for an OAR. *D_i_* is the dose of the *i*-th voxel; *D*_0_ is the objective dose; *p_TAR_* is the penalty weight of the OARs; *p_SOU_* is the dwell time modulation factor (DTMF); and *t_m,min_* is the smallest dwell time in the *m*-th channel.

The GBPO considered multiple targets and OARs, and each region of interest (ROI) had an objective dose. The last item in Equation (2) was provided to modulate the dwell time variance to meet the clinical needs.

### Test Cases

We divided the clinical test of the GBPO algorithm into two parts: the first part tested the optimization results of different applicators, which include a double ovoid applicator, a tandem-ring applicator, a multi-channel applicator, and a tandem-needles applicator. In the second part, we retrospectively studied 15 cervical cancer patients using the Fletcher applicator, and the average HR-CTV volume was 52.65 cm^3^ (minimum 36.03 cm^3^, maximum 80.45 cm^3^).

### Treatment Planning

The delineation of target and OARs was performed on an Oncentra V4.3 (Elekta AB, Stockholm, Sweden) TPS. The target was HR-CTV, and the OARs included the bladder, rectum, and sigmoid. The dose prescription was 6 Gy.

For each patient, we compared the following three plans: the IPSA plan, the Graphic plan, and the GBPO plan. For all plans, the source step size was 0.25 cm, and the dose calculation grid resolution was 0.1 cm x 0.1 cm x 0.1 cm. Since the optimization results were affected by the dwell point number and dwell position, the three plans used the same dwell point number and dwell position.

The IPSA plan automatically determined dwell positions based on the reference target. The DTDC value affects the optimization result (13), and we set it to 0.4 for all IPSA plans in this study.

We changed the dwell time of each dwell position to 1 before the Graphic optimization, and then a physicist manually dragged the isodose line to achieve a desirable dose distribution. The quality of the Graphic plan heavily depends on the clinical experience of the physicist. In order to improve the quality of the Graphic plan, the planning was performed by an experienced physicist who has worked in the brachytherapy department for more than 5 years.

For the GBPO plan, we set the initial dwell time of each dwell position to 1. The minimum value of the dwell time in the GBPO iteration process was set to 0.000001, which ensures the non-negativity of the dwell time during the optimization process. All GBPO plans were iterated 100 times. **Table 1** gives the optimization objectives for studying the relationship between the DTMF and the dwell time standard deviation (DTSD), as well as the initial objectives of the GBPO plans used for the comparison with the IPSA plans. In the dosimetric comparison process, if the optimization result of a GBPO plan was not satisfactory, we adjusted the initial objectives until obtaining a satisfying result. We set the DTMF to 10 for all GBPO plans, based on the results given in **Figure 2**.

**Table 1:** The optimization objectives for GBPO plans (Dose unit: Gy).

ROI	Weight	MIN	MAX	Weight
HR-CTV	100	6.0	–	–
Bladder	–	–	4.5	50
Rectum	–	–	4.5	80
Sigmoid	–	–	4.5	80

“-” means that this parameter was not used in the optimization.

### Plan Evaluation

We assessed the plan quality of GBPO by isodose lines for the 4 patients using different applicators. For the 15 patients using the Fletcher applicator, dose volume histogram (DVH) parameters were used to evaluate the dosimetric difference between the GBPO plans and the IPSA plans, as well as the GBPO plans and the Graphic plans. We defined *D_x_*_%_ as the dose expressed in Gy that received by *x*% of the total volume, *V_y_*_%_ as the volume expressed in percentage that received *y*% of the prescribed dose, and *D_zcc_* as the dose expressed in Gy that received by *z* *cm* ^3^ volume. The DVH parameters for HR-CTV were *D*_100%_ and *V*_150%_, and for the OARs, they were *D*_0.1_ *_cc_*、*D*_1_ *_cc_*and *D*_2_ *_cc_* (14). All plans were normalized to HR-CTV *D*_90%_ =6Gy. To evaluate the dosimetric parameters mentioned above, the SPSS 19.0 software (IBM, Armonk, NY) was used for statistical analyses. We conducted paired, a two-sided Wilcoxon signed-rank test to compare the dose distributions between the GBPO plans and the IPSA plans, as well as the GBPO plans and the Graphic plans. A *P* value less than 0.05 was considered statistically significant.

## Results

### Isodose Lines

**Figure 1:**
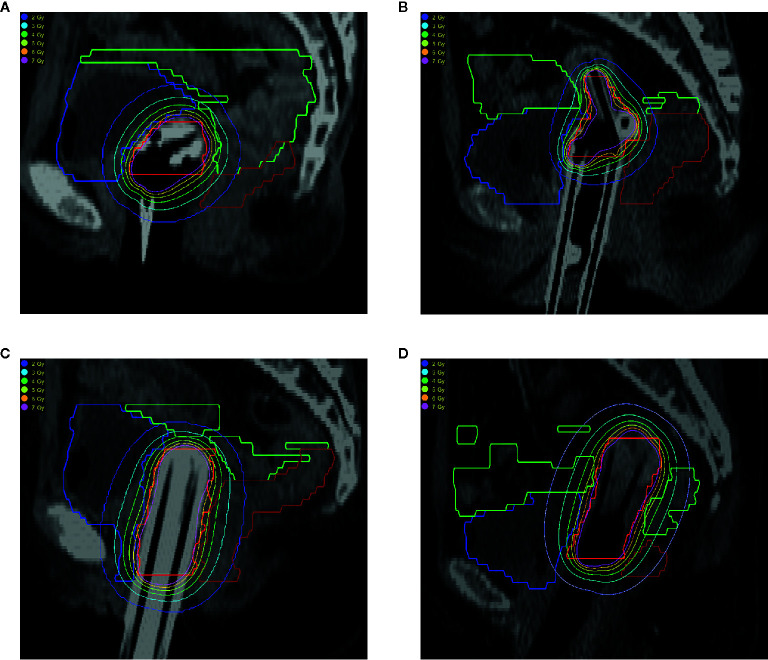
Isodose line of patients using different applicators. **(A)** Double ovoid applicator; **(B)** Tandem-ring applicator; **(C)** Multi-channel applicator; **(D)** Tandem-needles. The organs are HR-CTV (red), bladder (blue), rectum (brown), and sigmoid (green).

**Figure 1** shows the isodose lines optimized by the GBPO algorithm for the 4 patients using different applicators. The GBPO algorithm can generate conformable dose distributions for different applicators.

### Dwell Time Modulation Factor

**Figure 2:**
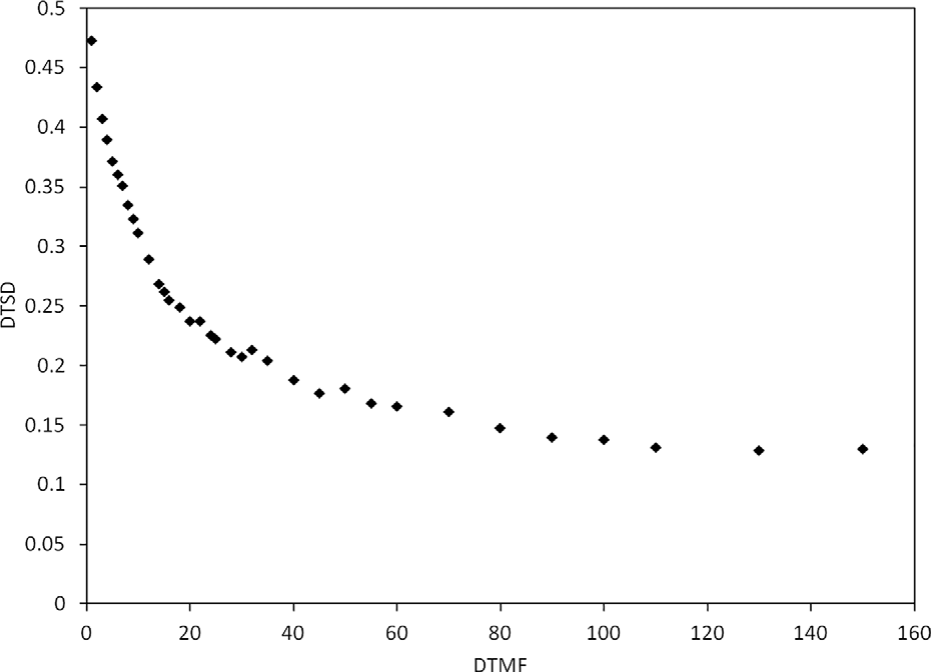
The dwell time standard deviation (DTSD) as a function of dwell time modulation factor (DTMF) for patients using the Fletcher applicator.

**Figure 2** illustrates the DTSD of the 15 patients using the Fletcher applicator optimized by the GBPO algorithm. When the DTMF is less than 20, the DTSD decreases quickly, but the DTSD has no remarkable change after the DTMF increased above 100. Therefore, in the planning optimization of cervical cancer, a DTMF value greater than 100 is not recommended when using the GBPO algorithm.

### Dosimetric Comparison

**Table 2** compares the DVH parameters of the target and OARs for the 15 patients. The difference in dosimetric parameters between the GBPO plans and the IPSA plans is not statistically significant (P > 0.05). The GBPO attains a similar plan quality as the IPSA. The GBPO plans has a higher D_100%_ (3.57 ± 0.36, 3.38 ± 0.34; P<0.01) and a lower V_150%_ (55.73 ± 4.06, 57.75 ± 3.79; P<0.01) than that of the Graphic plans. The differences in other DVH parameters are negligible between the GBPO plans and the Graphic plans.

**Table 2:** A dosimetric comparison for target and organs at risk.

Organ	Parameter	GBPO	IPSA	P value	Graphic	P value
HR-CTV	D_100%_	3.57 ± 0.36	3.54 ± 0.34	0.50	3.38 ± 0.34	0.00
	D_90%_	6.00 ± 0.00	6.00 ± 0.00	–	6.00 ± 0.00	–
	V_150%_	55.73 ± 4.06	56.43 ± 4.21	0.06	57.75 ± 3.79	0.00
Bladder	D_0.1cc_	6.08 ± 0.64	6.05 ± 0.75	0.60	6.15 ± 0.70	0.17
	D_1cc_	5.12 ± 0.53	5.11 ± 0.57	0.91	5.14 ± 0.56	0.18
	D_2cc_	4.74 ± 0.49	4.73 ± 0.53	0.29	4.76 ± 0.52	0.07
Rectum	D_0.1cc_	5.69 ± 0.90	5.76 ± 0.86	0.22	5.74 ± 0.87	0.33
	D_1cc_	4.63 ± 0.60	4.68 ± 0.60	0.21	4.70 ± 0.64	0.15
	D_2cc_	4.17 ± 0.57	4.20 ± 0.54	0.52	4.22 ± 0.61	0.16
Sigmoid	D_0.1cc_	2.77 ± 1.41	2.80 ± 1.34	0.51	2.69 ± 1.32	0.20
	D_1cc_	2.22 ± 1.21	2.25 ± 1.14	0.34	2.18 ± 1.10	0.50
	D_2cc_	2.00 ± 1.12	2.02 ± 1.04	0.51	1.96 ± 1.00	0.39

The dose unit is Gy, and the volume is a percentage volume. The data are listed as (mean ± standard deviation).

## Discussion

With the aid of imaging techniques such as CT and MRI, we can obtain an applicator position and ROIs three dimensionally. Knowing the applicator position and the source step size, we can also determine the dwell positions. A variety of algorithms have been developed to optimize the dwell time to achieve a desirable dose distribution (2–4, 15–18)_ENREF_11. In this study, we implemented a new inverse optimization algorithm, GBPO, to optimize the 3D brachytherapy dose based on patient anatomy and prescription dose. The patient data show that this algorithm achieves similar optimization results as compared with a commercial algorithm.

Uncertainties affect dose accuracy in high dose-rate brachytherapy (19). Regional hotspots should be avoided. Several studies have suggested using the dwell time modulation factor to address the issue of large dwell time variation (20, 21). In IPSA, the DTDC is a user-entered parameter that constrains the upper limit of a single dwell time relative to the average dwell time (4). The DTDC changes in the [0-1.0] range by a step of 0.1. When the DTDC is 0, it means that the optimization has no dwell time constraint, and the dwell time is the most uniform when it equals 1.0. The DTDC effectively reduces large dwell times, and neglects dwell times below the average value. The dwell time modulation factor of HIPO is DTGR, which avoids a large dwell time change between adjacent dwell locations, and eliminates the existence of large dwell times that may cause hotspots. Similar to the DTDC, the DTGR varies by a step size of 0.1 in the range of (0–1.0) (5). Increasing the DTGR value forces the optimizer to avoid situations where the dwell time is very long or very short. Since the DTGR considers the change of the adjacent dwell time, in places where there is no need to dwell, there may also be short dwell times if using DTGR.

The dwell time modulation principle of this algorithm is different from HIPO and IPSA. First, GBPO used the minimum dwell time in the objective function instead of the average value. The reason for this is that some of the dwell positions may not be suitable for dwell due to the OAR’s constraint, in which case the minimum dwell time is retained in GBPO. The purpose of adding a dwell time modulation item to the objective function is to make the larger dwell time shorter. Since the minimum dwell time is preserved, the DTSD will not be zero, even if the DTMF increases. Second, the GBPO does not normalize the maximum DTMF to 1. This study used the site of radical cervical cancer for testing, and did not consider other cancer sites. The normalized DTMF for one site may not be suitable for another site, so there are limitations in its application for other sites. In addition, using the same normalization method as IPSA and HIPO will make the modulation space smaller. There are only 11 values after normalization. Without normalization, we can change the DTMF value according to different clinical requirements.

There is currently a high incidence of cervical cancer (22). External beam radiation therapy combined with brachytherapy is the standard radiotherapy mode for cervical cancer (23). In our brachytherapy center, more than 90% patients have cervical cancer, which is why we selected cervical cancer patients as test cases. The Fletcher applicator is one of the most commonly used applicators for radical cervical cancer cases, it has 3 channels, and the optimization freedom is limited. However, compared with the IPSA plan, the GBPO obtained more favorable results, which gives us confidence this algorithm could be extended to other applicators and tumor sites.

For patients with radical cervical cancer, when the DTMF exceeds 100, the change in DTSD is not remarkable (**Figure 1**). Therefore, it is recommended to select the DTMF value within (0–100) for radial cervical cancers. It should be noted that **Figure 2** is based on the optimization parameters listed in **Table 1**. The relation of the DTSD and the DTMF may vary if the optimization parameter changes. Different sites may have a different DTMF- DTSD curve. Therefore, the DTMF- DTSD curve of other sites should be studied before using DTMF to determine an appropriate value suitable for other cancer sites. Testing the applicability of this algorithm to other cancers is a topic of our future work.

Dose optimization is a trial-and-error process, for a reverse optimization algorithm, the calculation speed is a factor that needs to be considered. The GBPO calculation is performed on a single central processor unit now, so the time required for GBPO is longer than that of the IPSA for the same test case. The GBPO running time for each case is about 2 to 5 min, depending on the quantity of dwell positions and dose points. In order to reduce the time spent on dose optimization, the GBPO needs parallel computing by MPI (Message Passing Interface) or CUDA (Compute Unified Device Architecture) technique. Parallel computing is the work we are currently doing.

## Conclusions

In this paper, we investigated a new inverse optimization algorithm, GBPO, for 3D brachytherapy, including a new dwell time modulation method. For a commonly used applicator in cervical cancer, this algorithm achieved similar results as compared with the IPSA optimization method. The GBPO algorithm could be integrated into a 3D brachytherapy TPS after more cancer sites are studied.

## Data Availability Statement

The original contributions presented in the study are included in the article/supplementary material; further inquiries can be directed to the corresponding author.

## Ethics Statement

The studies involving human participants were reviewed and approved by Sichuan Cancer Hospital Ethics Committee. The patients/participants provided their written informed consent to participate in this study. Written informed consent was obtained from the individual(s) for the publication of any potentially identifiable images or data included in this article.

## Author Contributions

XW, PW, JLi: Study design. XW, BT, SK, LL, JLa: Data collection and data analysis. XW, PW, LO, JLi: Manuscript preparation, manuscript editing. QH, ZW, CG: Critical discussion of the manuscript. All authors contributed to the article and approved the submitted version.

## Funding

This research was supported by National Key Research and Development Project (No. 2016YFC0105103, No. 2017YFC0113100) and Chengdu Science and Technology Project (2019-YF09-00095-SN).

## Conflict of Interest

The authors declare that the research was conducted in the absence of any commercial or financial relationships that could be construed as a potential conflict of interest.
